# Up-Regulation of Fibroblast Growth Factor 23 Gene Expression in UMR106 Osteoblast-like Cells with Reduced Viability

**DOI:** 10.3390/cells11010040

**Published:** 2021-12-23

**Authors:** Sina Münz, Martina Feger, Bayram Edemir, Michael Föller

**Affiliations:** 1 Department of Physiology, University of Hohenheim, 70599 Stuttgart, Germany; s.muenz@uni-hohenheim.de (S.M.); martina.feger@uni-hohenheim.de (M.F.); 2 Department of Hematology and Oncology, Martin Luther University Halle-Wittenberg, 06120 Halle, Germany; bayram.edemir@uk-halle.de

**Keywords:** cisplatin, apoptosis, 1,25(OH)_2_D_3_, klotho, inflammation

## Abstract

Fibroblast growth factor 23 (FGF23) controls vitamin D and phosphate homeostasis in the kidney and has additional paracrine effects elsewhere. As a biomarker, its plasma concentration is associated with progression of inflammatory, renal, and cardiovascular diseases. Major stimuli of FGF23 synthesis include active vitamin D and inflammation. Antineoplastic chemotherapy treats cancer by inducing cellular damage ultimately favoring cell death (apoptosis and necrosis) and causing inflammation. Our study explored whether chemotherapeutics and other apoptosis inducers impact on *Fgf23* expression. Experiments were performed in osteoblast-like UMR106 cells, *Fgf23* gene expression and protein synthesis were determined by qRT-PCR and ELISA, respectively. Viability was assessed by MTT assay and NFκB activity by Western Blotting. Antineoplastic drugs cisplatin and doxorubicin as well as apoptosis inducers procaspase-activating compound 1 (PAC-1), a caspase 3 activator, and serum depletion up-regulated *Fgf23* transcripts while reducing cell proliferation and viability. The effect of cisplatin on *Fgf23* transcription was paralleled by *Il-6* up-regulation and NFκB activation and attenuated by Il-6 and NFκB signaling inhibitors. To conclude, cell viability-decreasing chemotherapeutics as well as apoptosis stimulants PAC-1 and serum depletion up-regulate *Fgf23* gene expression. At least in part, Il-6 and NFκB may contribute to this effect.

## 1. Introduction

Cells that make up bone, osteoblasts, and osteocytes produce fibroblast growth factor 23 (FGF23), a protein with classical endocrine, but also paracrine effects [1,2]. As a hormone, it targets renal sodium phosphate co-transporter NaP_i_IIa, the main phosphate transporter of the proximal tubule, thereby enhancing urinary elimination of phosphate [3]. Moreover, FGF23 down-regulates *CYP27B1*, the renal key enzyme for the activation of vitamin D [4]. Therefore, FGF23 lowers the plasma concentration of active vitamin D (1,25(OH)_2_D_3_), which itself is a major regulator of phosphate metabolism [5]. Further endocrine effects of FGF23 are effective in the parathyroid gland, where FGF23 reduces parathyroid hormone (PTH) expression and secretion [6]. These classical endocrine effects require a complex of a FGF receptor (FGFR) and co-receptor αKlotho, a transmembrane protein with high expression in the kidney and parathyroid gland [7,8,9]. A certain motif with FGF23-independent endocrine and paracrine effects can be released from αKlotho upon cleavage, called soluble Klotho (sKl) [7,10]. The correct interplay of FGF23 and αKlotho in the regulation of phosphate and vitamin D metabolism is critical: mice deficient for FGF23 or αKlotho age rapidly and exhibit premature aging-associated diseases with death at young age, whereas overexpression of αKlotho extends life span by about 30% [11,12,13].

Elevations of the plasma FGF23 concentration were found in many clinical conditions including renal [14,15], cardiovascular [16,17,18,19], and inflammatory diseases [20]. Particularly in chronic kidney disease (CKD), changes in FGF23 level can be detected very early and correlate with outcome [21].

For this reason, regulation of FGF23 production and secretion is of high interest. Regulators of FGF23 thus far disclosed include dietary phosphate [22], PTH [23], 1,25(OH)_2_D_3_ [24], insulin [25], erythropoietin [26], or inflammation [27]. Pro-inflammatory cytokines such as interleukin-6 (Il-6) [28], tumor necrosis factor alpha (TNFα) [29] or transcription factor complex NFκB (nuclear factor kappa-light-chain-enhancer of activated B-cells) are major drivers of *FGF23* expression [30].

For most malignancies, chemotherapy is part of therapy either at certain stages, or along with other approaches (e.g., surgery, radiation) [31]. Common chemotherapeutics are cytotoxic drugs damaging cells and inducing apoptosis [32]. Among them are anthracyclines (e.g., doxorubicin) that intercalate with DNA [33] or platinum derivatives (e.g., cisplatin) inhibiting DNA replication by DNA cross-linking [34]. Initiation of apoptosis ultimately results in the activation of executioner caspase 3, which can directly be activated by procaspase-activating compound 1 (PAC-1) [35]. Lack of growth factors also induces apoptosis, which can be accomplished by serum depletion in cell culture [36].

Chemotherapeutics induce strong inflammation [37]. Moreover, chemotherapy with platinum derivatives is nephrotoxic [38] whereas anthracyclines are cardiotoxic [39]. In view of the strong *FGF23* expression-inducing properties of pro-inflammatory pathways [27] and kidney or cardiovascular damage elevating FGF23 plasma levels, we hypothesized that chemotherapeutic drugs may up-regulate *FGF23* expression. This may result in higher FGF23 plasma levels in patients undergoing chemotherapy and may have clinical relevance. Therefore, this study aimed to explore the impact of antineoplastic drugs and apoptosis stimulants on FGF23 in vitro. Moreover, we aimed to elucidate underlying mechanisms.

## 2. Materials and Methods

### 2.1. Cell Culture

Rat osteoblast-like UMR106 cells (CRL-1661; ATCC, Manassas, VA, USA) were cultured in Dulbecco’s Modified Eagle Medium (DMEM) containing 25 mM glucose and 1 mM pyruvate (Gibco, Life Technologies, Thermo Scientific, Darmstadt, Germany), supplemented with 10% fetal bovine serum (FBS; Gibco, Life Technologies), 100 U/mL penicillin, and 100 µg/mL streptomycin (Gibco, Life Technologies) at 5% CO_2_ and 37 °C. Serum depletion was accomplished for 24 h or 48 h by incubating the cells in culture medium with 1% or 0% FBS and additional 10 nM 1,25(OH)_2_D_3_ (Tocris, Bioscience, Bristol, UK) to enhance *Fgf23* expression [40]. Cells were seeded into 6-well plates (Greiner Bio-One, Frickenhausen, Germany) for 24 h. Subsequently, cisplatin, PAC-1 or doxorubicin (all from Tocris Bioscience) were added at the indicated concentrations for 24 or 48 h or the FBS concentration was reduced as described above. Il-6 signaling was blocked through gp130 inhibitor SC144 (1 µM, Tocris Bioscience). NFκB inhibitors withaferin A (Tocris Bioscience) and wogonin (Merck, Darmstadt, Germany) were used at concentration of 500 nM and 100 µM, respectively, where indicated.

To study cell proliferation, cells were trypsinized after 24 h or 48 h, respectively, and counted on a Neubauer hemocytometer.

### 2.2. Quantitative Real Time PCR

Total RNA was isolated from UMR106 cells using RNA-Solv reagent (Omega Bio-Tek, Norcross, GA, USA), and 1.2 µg thereof was used for cDNA synthesis with the GoScript Reverse Transcription System and random primers (Promega, Mannheim, Germany) on a Biometra TAdvanced thermal cycler (Analytik Jena, Jena, Germany).

Two µL cDNA was subjected to quantitative real-time PCR (qRT-PCR) with the CFX Connect Real-Time PCR Detection System (Bio-Rad, Feldkirchen, Germany). The reaction mix contained 0.25 µM (*Fgf23*) or 0.5 µM (*TATA-binding protein (Tbp), Il6, Rela*) of each primer, 10 µL GoTaq qPCR Master Mix (Promega), and sterile water to 18 µL per sample. 

The following rat primers were used (5′→3′): 

*Fgf23*: TAGAGCCTATTCAGACACTTC and CATCAGGGCACTGTAGATAG; 

*Tbp*: ACTCCTGCCACACCAGCC and GGTCAAGTTTACAGCCAAGATTCA;

*Il6*: CAGAGTCATTCAGAGCAATAC and CTTTCAAGATGAGTTGGATGG;

*Rela*: GCACCCCACCATCAAGATCAA and CTTGCTCCAGGTCTCGCTTC.

*Fgf23, Il6* and *Rela* transcript levels were normalized to transcript levels of housekeeping gene *Tbp* [41,42,43] and evaluated with the 2^-ΔCt^ method. 

### 2.3. Viability Assay (MTT Assay)

Cells were seeded into 96-well plates and treated for 24 or 48 h with cytostatic agents cisplatin or doxorubicin or apoptosis inducers PAC-1 or serum deprivation. Subsequently, cells were incubated with 0.5 mg/mL 3-[4,5-dimethylthiazol-2-yl]-2,5-diphenyltetrazolium bromide (MTT; Sigma-Aldrich, Schnelldorf, Germany) for 1 h. Next, MTT solution was removed, cells lyzed in dimethyl sulfoxide (DMSO; AppliChem, Darmstadt, Germany), and absorption measured at 550 nm and 690 nm (reference) on a FluoStar Omega plate reader (BMG Labtech, Ortenberg, Germany). Results are given as percentage of viable cells compared to control cells.

### 2.4. Enzyme Linked Immunosorbent Assay (ELISA)

Cell culture supernatants were collected and concentrated using Vivaspin^®^ 2 ultrafiltration columns (Sartorius, Göttingen, Germany). C-terminal FGF23 protein concentration was then determined by ELISA according to the manufacturer’s protocol (Immutopics, San Clemente, CA, USA). 

### 2.5. Western Blot

UMR106 cells were seeded into T25 cell culture flasks (Greiner Bio-One) and cultured for 24 h under standard conditions, then treated with 10 µM cisplatin or vehicle for another 24 h. Next, cells were lyzed using RIPA buffer (Cell Signaling Technology, Frankfurt, Germany) supplemented with protease and phosphatase inhibitor cocktail and EDTA (Halt, Thermo Scientific), total protein concentration measured by Bradford assay (Bio-Rad), and 30 µg of total protein subjected to 10% SDS-PAGE and standard Western Blotting. The following antibodies were used: anti-phospho-p65-NFκB (Ser536; 93H1), anti-GAPDH (D16H11), and anti-rabbit IgG HRP-linked antibody (all from Cell Signaling Technology). For visualization, membranes were incubated for 2 min with Westar Nova 2.0 (GAPDH) or Westar Supernova (phospho-p65-NFκB) ECL substrate (both from Cyanagen, Bologna, Italy). The densitometrical analysis was performed on a C-Digit^®^ Blot scanner (Li-Cor, Lincoln, NE, USA) and phospho-p65-NFκB bands were normalized to GAPDH bands using the Image Studio™ software (Li-Cor).

### 2.6. Statistics

Data are shown as arithmetic means ±  standard error of the mean (SEM) with *n* representing the number of independent experiments. Normal distribution was tested using Shapiro–Wilk normality test. Effects on cell number and viability and western blots were analyzed with one-sample *t*-test or one-sample Wilcoxon signed rank test, respectively. Two groups were analyzed with student’s *t*-test, Welch’s test, or Mann–Whitney U test. More than two groups were analyzed with one-way analysis of variance (ANOVA) followed by Dunnett’s multiple comparison test, Dunnett T3 test, or with non-parametric Kruskal–Wallis test followed by Dunn–Bonferroni post hoc test. Differences were considered significant if *p* < 0.05. Statistics were made using IBM SPSS Statistics (Version 27.0; Armonk, NY, USA).

## 3. Results

**Figure 1:**
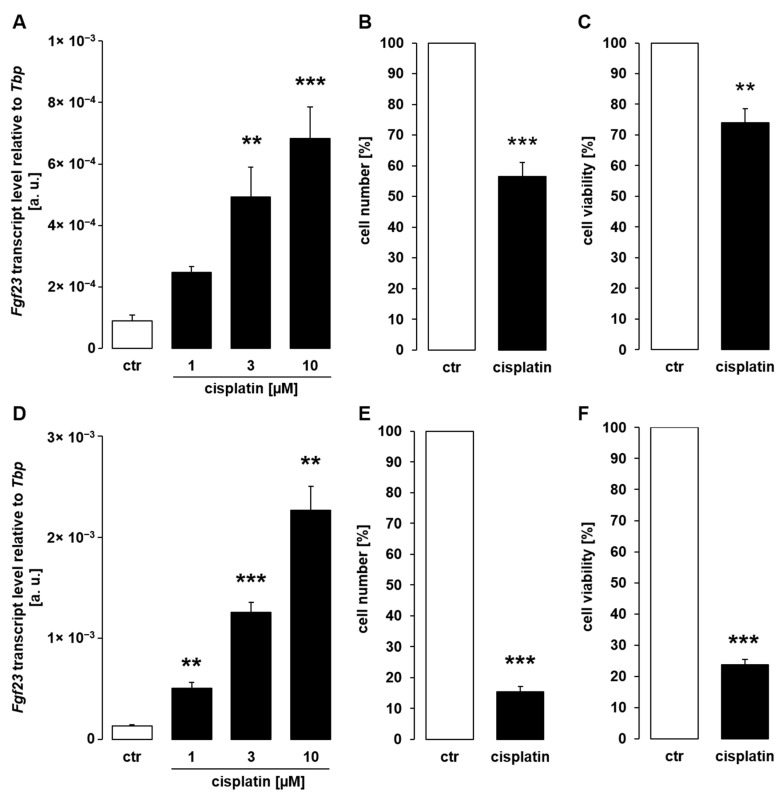
Cisplatin induced fibroblast growth factor 23 (*Fgf23*) expression in UMR106 cells. (**A**,**D**): Arithmetic means ± SEM of *Fgf23* transcript abundance relative to *TATA-binding protein* (*Tbp*) in UMR106 cells treated with vehicle control (ctr) or cisplatin at the indicated concentrations for 24 h ((**A**), *n* = 6; ANOVA followed by Dunnett’s multiple comparison test) or 48 h ((**D**), *n* = 6; one-way ANOVA followed by Dunnett T3 multiple comparison test). (**B**–**F**): Arithmetic means ± SEM of the number ((**B**); *n* = 7; one-sample *t*-test; (**E**), *n* = 6; one-sample *t*-test) or viability ((**C**); *n* = 6; one-sample *t*-test; (**F**); *n* = 5; one-sample *t*-test) of UMR106 cells treated without or with 10 µM cisplatin for 24 h (**B**,**C**) or 48 h (**E**,**F**). All values are relative to the respective values of vehicle-treated cells. ** *p* < 0.01, *** *p* < 0.001 indicate significant difference from control cells. a. u., arbitrary units; ctr, control.

To investigate whether chemotherapeutics impact on *Fgf23* expression, we performed experiments in UMR106 osteoblast-like cells. In a first series of experiments, the cells were treated with platinum derivative cisplatin, an antineoplastic drug used in the treatment of a variety of malignancies, and *Fgf23* transcript levels were determined by qRT-PCR. As demonstrated in Figure 1A, cisplatin enhanced *Fgf23* gene expression in UMR106 cells in a dose-dependent manner within 24 h. By the same token, exposure to cisplatin reduced number (Figure 1B) and viability (Figure 1C) of UMR106 cells following a 24-h exposure.

To check whether upregulation of *Fgf23* gene expression is a stress reaction only observable at 24 h, we extended exposure time in a further series of experiments. According to Figure 1D, also a 48-h exposure of UMR106 cells resulted in dose-dependent upregulation of *Fgf23* gene expression. Cell number (Figure 1E) and viability (Figure 1F), however, were more strongly reduced upon a 48-h exposure to cisplatin compared to a 24-h incubation (Figure 1B,C).

**Figure 2:**
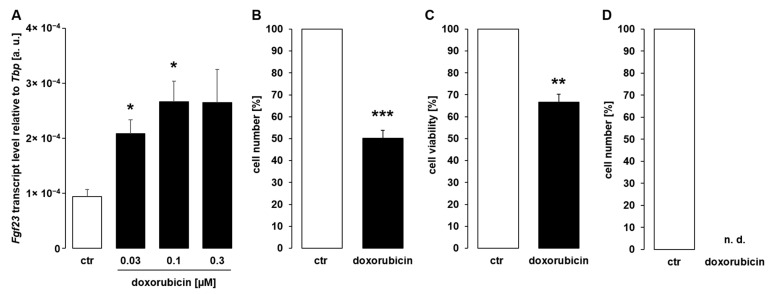
Doxorubicin enhanced *Fgf23* expression in UMR106 cells. (**A**): Arithmetic means ± SEM of *Fgf23* transcript abundance relative to *Tbp* in UMR106 cells treated for 24 h with vehicle control (ctr) or doxorubicin at the indicated concentrations (*n* = 6; one-way ANOVA followed by Dunnett T3 multiple comparison test). (**B**–**D**): Arithmetic means ± SEM of the number ((**B**); *n* = 5; one-sample *t*-test; (**D**); *n* = 4) or viability ((**C**); *n* = 5; one-sample *t*-test) of UMR106 cells treated without or with 0.1 µM doxorubicin for 24 h (**B**,**C**) or 48 h (**D**). All values are relative to the respective values of vehicle-treated cells. * *p* < 0.05, ** *p* < 0.01, *** *p* < 0.001 indicate significant difference from vehicle-treated cells. a.u., arbitrary units; ctr, control; n.d., not detectable.

The next series of experiments was carried out to investigate whether anthracyclines, chemotherapeutic drugs that inhibit topoisomerase and intercalate with DNA [33], are similarly capable of inducing *Fgf23* gene expression. UMR106 cells exposed to doxorubicin (0.03–0.3 µM) for 24 h exhibited enhanced *Fgf23* gene expression in a dose-dependent manner (Figure 2A). Similar to cisplatin, doxorubicin also compromised cell proliferation (Figure 2B) and viability (Figure 2C). Again, we tested whether a longer exposure similarly up-regulated *Fgf23*. As a result, incubation of UMR106 cells with doxorubicin for 48 h killed virtually all cells (Figure 2D). Hence, *Fgf23* transcripts were not detectable after 48 h.

**Figure 3:**
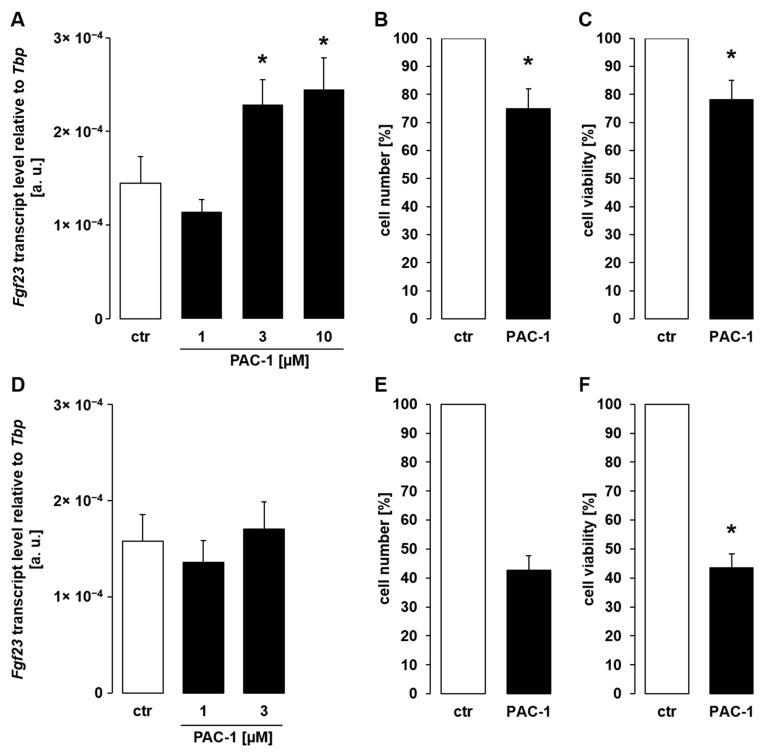
Procaspase-activating compound 1 (PAC-1) stimulated *Fgf23* expression in UMR106 cells. (**A**,**D**): Arithmetic means ± SEM of *Fgf23* transcript abundance relative to *Tbp* in UMR106 cells treated for 24 h ((**A**); *n* = 10; Kruskal–Wallis test followed by Dunn–Bonferroni test) or 48 h ((**D**); *n* = 10; one-way ANOVA) with vehicle control (ctr) or PAC-1 at the indicated concentrations. (**B**–**F**): Arithmetic means ± SEM of the number ((**B**); *n* = 5; one-sample *t*-test; (**E**); *n* = 4; one-sample Wilcoxon signed rank test) or viability ((**C**); *n* = 5; one-sample Wilcoxon signed rank test; (**F**); *n* = 5; one-sample Wilcoxon signed rank test) of UMR106 cells treated with vehicle control (ctr) or 3 µM PAC-1 for 24 h (**B**,**C**) or 48 h (**E**,**F**). All values are relative to the respective values of control-treated cells. * *p* < 0.05 indicates significant difference from vehicle-treated cells. a. u., arbitrary units; ctr, control.

Our results indicate that cytotoxic reagents up-regulate *Fgf23* gene expression in UMR106 cells. In order to test whether this effect is mimicked by direct stimulation of apoptosis, PAC-1, an activator of apoptosis-initiating executioner caspase 3, was applied. As demonstrated in Figure 3A, similar to chemotherapeutics, PAC-1 dose-dependently up-regulated *Fgf23* gene expression in UMR106 cells within 24 h. This effect was paralleled by compromised cell proliferation (Figure 3B) and viability (Figure 3C), as well. A 48-h exposure to PAC-1 did not significantly modify *Fgf23* transcripts in UMR106 cells (Figure 3D) while suppressing cell proliferation (Figure 3E) and viability (Figure 3F).

**Figure 4:**
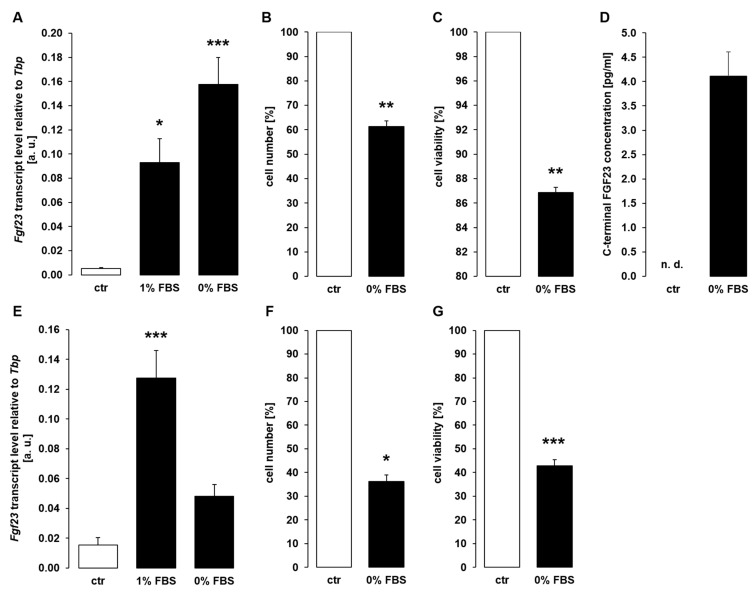
Serum depletion induced *Fgf23* expression and secretion in osteoblast-like UMR106 cells. (**A**): Arithmetic means ± SEM of *Fgf23* transcript level relative to *Tbp* in UMR106 cells incubated for 24 h in medium containing 10% (ctr), 1%, or 0% fetal bovine serum (FBS) (*n* = 6; Kruskal–Wallis test followed by Dunn–Bonferroni post hoc test). (**B**,**C**): Arithmetic means ± SEM of the number ((**B**); *n* = 4; one-sample *t*-test) or viability ((**C**); *n* = 4; one-sample *t*-test) of UMR106 cells incubated for 24 h without FBS relative to the respective value of cells incubated in 10% FBS. (**D**): Arithmetic means ± SEM of C-terminal FGF23 protein concentration in the supernatant of UMR106 cells incubated with 10% FBS (ctr) or without FBS for 24 h (*n* = 7). (**E**): Arithmetic means ± SEM of *Fgf23* mRNA levels relative to *Tbp* levels of UMR106 cells treated for 48 h with medium containing 10% (ctr), 1%, or 0% FBS (*n* = 7; Kruskal–Wallis followed by Dunn–Bonferroni test). (**F**,**G**): Arithmetic means ± SEM of cell number ((**F**), *n* = 6; one-sample Wilcoxon signed rank test) or cell viability ((**G**), *n* = 5; one-sample *t*-test) of UMR106 cells incubated in culture medium with 10% FBS (ctr) or without FBS for 48 h. In all experiments, cell culture medium contained 10 nM 1,25(OH)_2_D_3_. * *p* < 0.05, ** *p* < 0.01, *** p < 0.001 indicate significant difference from control cells. a. u., arbitrary units; ctr, control; n. d., not detectable.

Since direct apoptosis inducer PAC-1 enhanced *Fgf23* gene expression in UMR106 cells, we performed a further series of experiments to study whether another stimulant of apoptosis, depletion of cell growth factors, also affects *Fgf23* transcription. To this end, we incubated UMR106 cells for 24 h under normal conditions (10% FBS), under conditions of reduced FBS (1%), and without FBS in the presence of 10 nM 1,25(OH)_2_D_3_. Serum depletion resulted in a strong up-regulation of *Fgf23* gene expression (Figure 4A). Again, the effect was paralleled by decreased proliferation (Figure 4B) and viability (Figure 4C) of UMR106 cells. The stimulatory effect of serum depletion on *Fgf23* transcripts was followed by enhanced secretion of C-terminal FGF23 protein into the cell culture supernatant (Figure 4D). Also, 48 h serum depletion up-regulated *Fgf23* gene expression (Figure 4E), an effect again paralleled by reduced proliferation (Figure 4F) and viability (Figure 4G). 

**Figure 5:**
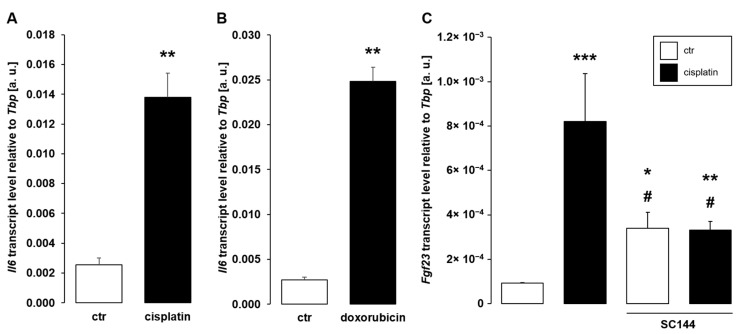
Interleukin-6 (IL-6) signaling inhibitor SC144 attenuated cisplatin-induced *Fgf23* gene expression in UMR106 cells. (**A**,**B**): Arithmetic means ± SEM of interleukin-6 (*Il6*) mRNA levels relative to *Tbp* in UMR106 cells treated without (ctr) or with 10 µM cisplatin ((**A**), *n* = 6; Welch’s test) or 0.3 µM doxorubicin ((**B**), *n* = 6; Mann–Whitney U test) for 24 h. (**C**): Arithmetic means ± SEM of *Fgf23* transcript levels relative to *Tbp* in UMR106 cells treated without (ctr) or with 10 µM cisplatin in the presence or absence of 1 µM Il-6 signaling inhibitor SC144 (*n* = 9; Kruskal–Wallis followed by Dunn–Bonferroni test) for 24 h. * *p* < 0.05, ** *p* < 0.01, *** *p* < 0.001 indicate significant differences from vehicle-treated cells (1st bar); # *p* < 0.05 indicates significant difference from absence of SC144 (2nd bar vs. 4th bar). a. u., arbitrary units; ctr, control.

Pro-inflammatory cytokines including Il-6 are major stimuli of *Fgf23* expression, and chemotherapy has been shown to enhance inflammation [44]. A further series of experiments, therefore, aimed to explore the role of Il-6 for antineoplastic drug-dependent up-regulation of *Fgf23*. As illustrated in Figure 5, a 24-h exposure of UMR106 cells to 10 µM cisplatin (Figure 5A) or 0.3 µM doxorubicin (Figure 5B) readily stimulated *Il6* gene expression. Importantly, SC144, an Il-6 signaling inhibitor blocking gp130, significantly attenuated cisplatin-induced *Fgf23* transcription (Figure 5C)

**Figure 6:**
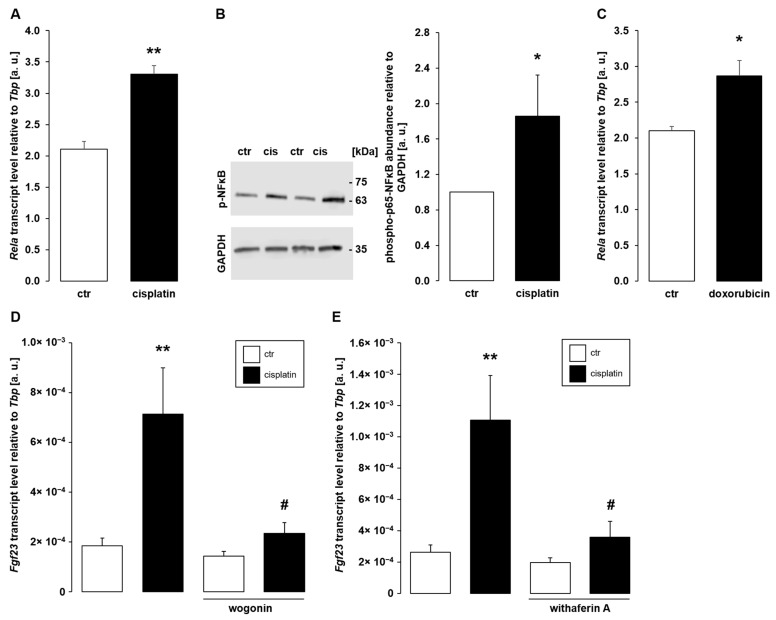
The contribution of NFκB to the *Fgf23* effect of cisplatin. (**A**): Arithmetic means ± SEM of NFκB subunit p65 (*Rela*) gene expression relative to *Tbp* in UMR106 cells incubated without (ctr) or with 10 µM cisplatin for 24 h (*n* = 4; student’s *t*-test). (**B**): Left panel: Original Western Blot demonstrating the abundance of phospho-p65-NFκB and GAPDH in UMR106 cells treated with (cis) or without (ctr) 10 µM cisplatin for 24 h. Right panel: Arithmetic means ± SEM of phospho-p65-NFκB relative to GAPDH abundance (*n* = 8; one-sample Wilcoxon signed rank test). (**C**): Arithmetic means ± SEM of *Rela* expression relative to *Tbp* in UMR106 cells incubated for 24 h without (ctr) or with 0.3 µM doxorubicin (*n* = 4; student’s *t*-test). (**D**,**E**): Arithmetic means ± SEM of *Fgf23* transcript abundance relative to *Tbp* in UMR106 cells treated for 24 h with vehicle control (ctr, white bars) or 3 µM cisplatin (black bars) in the presence or absence of 100 µM wogonin ((**D**); *n* = 9; Kruskal–Wallis test followed by Dunn–Bonferroni test) or 500 nM withaferin A ((**E**); *n* = 9; Kruskal–Wallis test followed by Dunn–Bonferroni test). * *p* < 0.05, ** *p* < 0.01 indicate significant difference from vehicle-treated cells (1st bar). # *p* < 0.05 indicates significant difference from the absence of NFκB inhibitors wogonin and withaferin A, respectively (2^nd^ bar vs. 4^th^ bar). a. u., arbitrary units; ctr, control.

Downstream signaling of pro-inflammatory stimuli may eventually result in the activation of transcription factor complex NFκB, an important driver of FGF23 production [30]. Further experiments, therefore, focused on the involvement of NFκB in the stimulation of *Fgf23* by cisplatin. Within 24 h, treatment of UMR106 cells with 10 µM cisplatin resulted in enhanced *Rela* expression, the gene encoding p65 subunit of NFκB (Figure 6A). As detected by Western Blotting, cisplatin (10 µM, 24 h) significantly stimulated phosphorylation of p65 (Figure 6B). Moreover, treatment with doxorubicin (0.3 µM, 24 h) enhanced *Rela* expression (Figure 6C). Hence, cisplatin and doxorubicin induced NFκB activity in UMR106 cells. A last series of experiments explored whether NFκB activity is required for the effect of cisplatin on *Fgf23*. To this end, UMR106 cells were treated with and without cisplatin and NFκB inhibitors wogonin or withaferin A for 24 h. As depicted in Figure 6D, wogonin significantly attenuated the cisplatin-induced effect on *Fgf23* gene expression. Similarly, withaferin A blunted cisplatin-induced up-regulation of *Fgf23* (Figure 6E).

## 4. Discussion

According to our study, two cytotoxic drugs with different cellular targets used in the treatment of several malignancies as well as apoptosis inducers PAC-1 and serum depletion stimulated *Fgf23* gene expression in UMR106 osteoblast-like cells within 24 h. The effect was paralleled by a reduction in cell viability and proliferation as deduced from cell number.

UMR106 osteoblast-like cells were chosen for our study because under physiological conditions, bone is the major site of FGF23 production [45] and these cells are a versatile tool employed in many studies to unravel the regulation of FGF23 [25,46,47,48,49].

Incubation of UMR106 cells with cisplatin or in serum-depleted medium for 48 h also resulted in enhanced *Fgf23* expression. Prolonged incubation with doxorubicin, however, killed all cells. In contrast to 24 h, 48-h exposure of the cells to PAC-1 did not significantly modify *Fgf23* expression, possibly because PAC-1-dependent apoptosis induction occurs much earlier and late apoptotic cells cannot up-regulate *Fgf23* gene expression any longer. 

Cisplatin, doxorubicin, PAC-1 as well as serum depletion have in common that they cause cellular damage reducing cell number and viability, which may ultimately result in cell death. Cisplatin is effective by interfering with DNA replication [50], doxorubicin inhibits topoisomerase and intercalates with DNA [51], PAC-1 directly stimulates apoptotic cell death through executioner caspase 3 [35], whereas serum depletion favors apoptotic cell death due to lack of essential growth factors [36]. Although the mechanism of cell damage is different, the up-regulation of *Fgf23* gene expression is consistent for all four inducers of cellular injury. This important finding may point to a role of FGF23 in cellular stress, cell death, and survival. Indeed, FGF23-Klotho signaling favors cell proliferation and inhibits apoptosis, elicited by vitamin D, through phosphoinositide-3 kinase (PI3K) signaling [52]. Moreover, FGF23 exerts many effects through serum and glucocorticoid-dependent kinase 1 (SGK1) [53]. SGK1 is an important mediator of pro-survival signaling inhibiting apoptosis [54]. Moreover, in acute kidney injury (AKI), FGF23 has turned out to stimulate cell proliferation promoting regeneration of injured tubules through influencing SDF-1/CXCR4 signaling [55]. In tumor cells, namely prostate cancer, FGF23 similarly stimulates cell proliferation [56]. According to these studies, FGF23 has pro-survival/anti-apoptotic properties. Hence, up-regulation of FGF23 in cell stress as demonstrated in our study may help the cell activate pro-survival signaling. Alternatively, FGF23 may not only be a disease biomarker, but *Fgf23* gene expression may also indicate injury on cellular level or even serve as a marker for moribund cells. Definitely, further research is required to elucidate this.

In UMR106 cells, basal *Fgf23* expression is low unless the cells are pretreated with 1,25(OH)_2_D_3_ which strongly up-regulates *Fgf23* expression [24]. Therefore, it must be kept in mind that although *Fgf23* transcripts significantly increased upon treatment with cisplatin, doxorubicin, or PAC-1, yet the cellular FGF23 protein concentration remained below the detection limit of ELISA. Serum depletion experiments were accomplished in the presence of 10 nM 1,25(OH)_2_D_3_, hence, C-terminal FGF23 protein in the cell culture supernatant could be detected by ELISA and was significantly up-regulated in serum-depleted cells compared to control cells. 

Chemotherapy is known to induce inflammation [37]. We demonstrated that both cisplatin and doxorubicin induce pro-inflammatory cytokine Il-6 within 24 h. Importantly, Il-6 is a stimulator of FGF23 [28]. In line with this, Il-6 signaling inhibitor SC144 significantly blunted cisplatin-induced *Fgf23* gene expression. Moreover, expression and phosphorylation of NFκB subunit p65 were up-regulated by cisplatin. Accordingly, wogonin and withaferin A, inhibitors of NFκB, significantly blunted cisplatin-induced up-regulation of *Fgf23* expression. This is in line with the pivotal role of NFκB and generally inflammation for the stimulation of FGF23 production. Importantly, cisplatin is a powerful inducer of NFκB activity [57], which may also contribute to treatment resistance [58] or nephrotoxicity [59]. Doxorubicin also induces inflammation by activating NFκB [60,61]. Hence, it appears likely that chemotherapy-induced inflammation involving Il-6 and NFκB is a major contributor to the up-regulation of *Fgf23* expression. In our experiments, wogonin and withaferin A tended to decrease *Fgf23* transcript levels in untreated cells, a difference, however, not reaching statistical significance. Presumably, the effect of NFκB inhibition on Fgf23 is smaller in cells with low basal *Fgf23* expression in the absence of 1,25(OH)_2_D_3_ stimulation than in cells pre-treated with 1,25(OH)_2_D_3_ to up-regulate *Fgf23* expression [30].

Direct executioner caspase-3-activator PAC-1 also up-regulated *Fgf23* gene expression. The same holds true for serum depletion, which favors apoptosis through growth factor deficiency [62]. However, caspase 3 activation and subsequent apoptosis are rather associated with decreased NFκB activity and not with a pro-inflammatory response [63]. Hence, additional mechanisms elucidated by future studies can clearly be expected to be also involved in the up-regulation of *Fgf23* expression of injured cells. 

Taken together, the induction of cellular injury through cytotoxic drugs, serum depletion, or caspase 3 activation resulting in decreased proliferation and viability leads to the up-regulation of *Fgf23* gene expression. This effect can in part, but not fully, be explained by IL-6 up-regulation and NFκB activation.

## Data Availability

Not applicable.

## References

[1] Han Y., You X., Xing W., Zhang Z., Zou W. (2018). Paracrine and endocrine actions of bone—The functions of secretory proteins from osteoblasts, osteocytes, and osteoclasts. Bone Res..

[2] Leifheit-Nestler M., Haffner D. (2018). Paracrine Effects of FGF23 on the Heart. Front. Endocrinol..

[3] Hu M.C., Shi M., Moe O.W. (2019). Role of αKlotho and FGF23 in regulation of type II Na-dependent phosphate co-transporters. Pflug. Arch..

[4] Chanakul A., Zhang M.Y.H., Louw A., Armbrecht H.J., Miller W.L., Portale A.A., Perwad F. (2013). FGF-23 Regulates CYP27B1 Transcription in the Kidney and in Extra-Renal Tissues. PLoS ONE.

[5] Shimada T., Hasegawa H., Yamazaki Y., Muto T., Hino R., Takeuchi Y., Fujita T., Nakahara K., Fukumoto S., Yamashita T. (2004). FGF-23 Is a Potent Regulator of Vitamin D Metabolism and Phosphate Homeostasis. J. Bone Miner. Res..

[6] Ben-Dov I.Z., Galitzer H., Lavi-Moshayoff V., Goetz R., Kuro-o M., Mohammadi M., Sirkis R., Naveh-Many T., Silver J. (2007). The parathyroid is a target organ for FGF23 in rats. J. Clin. Investig..

[7] Hu M.C., Shiizaki K., Kuro-o M., Moe O.W. (2013). Fibroblast Growth Factor 23 and Klotho: Physiology and Pathophysiology of an Endocrine Network of Mineral Metabolism. Annu. Rev. Physiol..

[8] Mytych J., Sołek P., Będzińska A., Rusinek K., Warzybok A., Tabęcka-Łonczyńska A., Koziorowski M. (2020). Towards Age-Related Anti-Inflammatory Therapy: Klotho Suppresses Activation of ER and Golgi Stress Response in Senescent Monocytes. Cells.

[9] Rusinek K., Sołek P., Tabęcka-Łonczyńska A., Koziorowski M., Mytych J. (2020). Focus on the Role of Klotho Protein in Neuro-Immune Interactions in HT-22 Cells Upon LPS Stimulation. Cells.

[10] Imura A., Iwano A., Tohyama O., Tsuji Y., Nozaki K., Hashimoto N., Fujimori T., Nabeshima Y.-I. (2004). Secreted Klotho protein in sera and CSF: Implication for post-translational cleavage in release of Klotho protein from cell membrane. FEBS Lett..

[11] Kuro-o M., Matsumura Y., Aizawa H., Kawaguchi H., Suga T., Utsugi T., Ohyama Y., Kurabayashi M., Kaname T., Kume E. (1997). Mutation of the mouse klotho gene leads to a syndrome resembling ageing. Nature.

[12] Kurosu H., Yamamoto M., Clark J.D., Pastor J.V., Nandi A., Gurnani P., McGuinness O.P., Chikuda H., Yamaguchi M., Kawaguchi H. (2005). Suppression of aging in mice by the hormone Klotho. Science.

[13] Razzaque M.S., Lanske B. (2006). Hypervitaminosis D and premature aging: Lessons learned from Fgf23 and Klotho mutant mice. Trends Mol. Med..

[14] Wahl P., Wolf M., Kuro-o M. (2012). FGF23 in Chronic Kidney Disease. Endocrine FGFs and Klothos.

[15] Chu C., Elitok S., Zeng S., Xiong Y., Hocher C.-F., Hasan A.A., Krämer B.K., Hocher B. (2021). C-terminal and intact FGF23 in kidney transplant recipients and their associations with overall graft survival. BMC Nephrol..

[16] Xiao Y., Peng C., Huang W., Zhang J., Xia M., Zhang Y., Ling W. (2013). Circulating Fibroblast Growth Factor 23 Is Associated with Angiographic Severity and Extent of Coronary Artery Disease. PLoS ONE.

[17] Mirza M.A.I., Hansen T., Johansson L., Ahlström H., Larsson A., Lind L., Larsson T.E. (2009). Relationship between circulating FGF23 and total body atherosclerosis in the community. Nephrol. Dial. Transplant..

[18] Di Giuseppe R., Kühn T., Hirche F., Buijsse B., Dierkes J., Fritsche A., Kaaks R., Boeing H., Stangl G.I., Weikert C. (2015). Plasma fibroblast growth factor 23 and risk of cardiovascular disease: Results from the EPIC-Germany case-cohort study. Eur. J. Epidemiol..

[19] Figurek A., Rroji M., Spasovski G. (2021). The Complexity of FGF23 Effects on Cardiomyocytes in Normal and Uremic Milieu. Cells.

[20] Fitzpatrick E.A., Han X., Xiao Z., Quarles L.D. (2018). Role of Fibroblast Growth Factor-23 in Innate Immune Responses. Front. Endocrinol..

[21] Isakova T. (2012). Fibroblast growth factor 23 and adverse clinical outcomes in chronic kidney disease. Curr. Opin. Nephrol. Hypertens..

[22] Vervloet M.G., van Ittersum F.J., Büttler R.M., Heijboer A.C., Blankenstein M.A., ter Wee P.M. (2011). Effects of dietary phosphate and calcium intake on fibroblast growth factor-23. Clin. J. Am. Soc. Nephrol..

[23] Meir T., Durlacher K., Pan Z., Amir G., Richards W.G., Silver J., Naveh-Many T. (2014). Parathyroid hormone activates the orphan nuclear receptor Nurr1 to induce FGF23 transcription. Kidney Int..

[24] Masuyama R., Stockmans I., Torrekens S., van Looveren R., Maes C., Carmeliet P., Bouillon R., Carmeliet G. (2006). Vitamin D receptor in chondrocytes promotes osteoclastogenesis and regulates FGF23 production in osteoblasts. J. Clin. Investig..

[25] Bär L., Feger M., Fajol A., Klotz L.-O., Zeng S., Lang F., Hocher B., Föller M. (2018). Insulin suppresses the production of fibroblast growth factor 23 (FGF23). Proc. Natl. Acad. Sci. USA.

[26] Daryadel A., Bettoni C., Haider T., Imenez Silva P.H., Schnitzbauer U., Pastor-Arroyo E.M., Wenger R.H., Gassmann M., Wagner C.A. (2018). Erythropoietin stimulates fibroblast growth factor 23 (FGF23) in mice and men. Pflug. Arch..

[27] David V., Martin A., Isakova T., Spaulding C., Qi L., Ramirez V., Zumbrennen-Bullough K.B., Sun C.C., Lin H.Y., Babitt J.L. (2016). Inflammation and functional iron deficiency regulate fibroblast growth factor 23 production. Kidney Int..

[28] Durlacher-Betzer K., Hassan A., Levi R., Axelrod J., Silver J., Naveh-Many T. (2018). Interleukin-6 contributes to the increase in fibroblast growth factor 23 expression in acute and chronic kidney disease. Kidney Int..

[29] Glosse P., Fajol A., Hirche F., Feger M., Voelkl J., Lang F., Stangl G.I., Föller M. (2018). A high-fat diet stimulates fibroblast growth factor 23 formation in mice through TNFα upregulation. Nutr. Diabetes.

[30] Zhang B., Yan J., Umbach A.T., Fakhri H., Fajol A., Schmidt S., Salker M.S., Chen H., Alexander D., Spichtig D. (2016). NFκB-sensitive Orai1 expression in the regulation of FGF23 release. J. Mol. Med..

[31] Bold R.J., Termuhlen P.M., McConkey D.J. (1997). Apoptosis, cancer and cancer therapy. Surg. Oncol..

[32] Makin G., Hickman J.A. (2000). Apoptosis and cancer chemotherapy. Cell Tissue Res..

[33] Yang F., Kemp C.J., Henikoff S. (2015). Anthracyclines induce double-strand DNA breaks at active gene promoters. Mutat. Res. Fundam. Mol. Mech. Mutagen..

[34] Dasari S., Bernard Tchounwou P. (2014). Cisplatin in cancer therapy: Molecular mechanisms of action. Eur. J. Pharmacol..

[35] Peterson Q.P., Goode D.R., West D.C., Ramsey K.N., Lee J.J.Y., Hergenrother P.J. (2009). PAC-1 activates procaspase-3 in vitro through relief of zinc-mediated inhibition. J. Mol. Biol..

[36] Higuchi A., Shimmura S., Takeuchi T., Suematsu M., Tsubota K. (2006). Elucidation of apoptosis induced by serum deprivation in cultured conjunctival epithelial cells. Br. J. Ophthalmol..

[37] Vyas D., Laput G., Vyas A.K. (2014). Chemotherapy-enhanced inflammation may lead to the failure of therapy and metastasis. OncoTargets Ther..

[38] Ludwig T., Riethmüller C., Gekle M., Schwerdt G., Oberleithner H. (2004). Nephrotoxicity of platinum complexes is related to basolateral organic cation transport. Kidney Int..

[39] Volkova M., Russell R. (2011). Anthracycline cardiotoxicity: Prevalence, pathogenesis and treatment. Curr. Cardiol. Rev..

[40] Saini R.K., Kaneko I., Jurutka P.W., Forster R., Hsieh A., Hsieh J.-C., Haussler M.R., Whitfield G.K. (2013). 1,25-dihydroxyvitamin D(3) regulation of fibroblast growth factor-23 expression in bone cells: Evidence for primary and secondary mechanisms modulated by leptin and interleukin-6. Calcif. Tissue Int..

[41] González-Bermúdez L., Anglada T., Genescà A., Martín M., Terradas M. (2019). Identification of reference genes for RT-qPCR data normalisation in aging studies. Sci. Rep..

[42] Abuna R.P.F., Oliveira F.S., Ramos J.I.R., Lopes H.B., Freitas G.P., Souza A.T.P., Beloti M.M., Rosa A.L. (2018). Selection of reference genes for quantitative real-time polymerase chain reaction studies in rat osteoblasts. J. Cell. Physiol..

[43] Bär L., Hase P., Föller M. (2019). PKC regulates the production of fibroblast growth factor 23 (FGF23). PLoS ONE.

[44] Oflazoglu U., Alacacioglu A., Varol U., Kucukzeybek Y., Salman T., Onal H.T., Yilmaz H.E., Yildiz Y., Taskaynatan H., Saray S. (2020). The role of inflammation in adjuvant chemotherapy-induced sarcopenia (Izmir Oncology Group (IZOG) study). Support Care Cancer.

[45] Bonewald L.F., Wacker M.J. (2013). FGF23 production by osteocytes. Pediatr. Nephrol..

[46] Ma L., Gao M., Wu L., Zhao X., Mao H., Xing C. (2018). The suppressive effect of soluble Klotho on fibroblastic growth factor 23 synthesis in UMR-106 osteoblast-like cells. Cell Biol. Int..

[47] Vidal A., Rios R., Pineda C., Lopez I., Muñoz-Castañeda J.R., Rodriguez M., Aguilera-Tejero E., Raya A.I. (2020). Direct regulation of fibroblast growth factor 23 by energy intake through mTOR. Sci. Rep..

[48] Samadfam R., Richard C., Nguyen-Yamamoto L., Bolivar I., Goltzman D. (2009). Bone formation regulates circulating concentrations of fibroblast growth factor 23. Endocrinology.

[49] Takashi Y., Kosako H., Sawatsubashi S., Kinoshita Y., Ito N., Tsoumpra M.K., Nangaku M., Abe M., Matsuhisa M., Kato S. (2019). Activation of unliganded FGF receptor by extracellular phosphate potentiates proteolytic protection of FGF23 by its O-glycosylation. Proc. Natl. Acad. Sci. USA.

[50] Siddik Z.H. (2003). Cisplatin: Mode of cytotoxic action and molecular basis of resistance. Oncogene.

[51] Wang C.-W., Chen C.-L., Wang C.-K., Chang Y.-J., Jian J.-Y., Lin C.-S., Tai C.-J., Tai C.-J. (2015). Cisplatin-, Doxorubicin-, and Docetaxel-Induced Cell Death Promoted by the Aqueous Extract of Solanum nigrum in Human Ovarian Carcinoma Cells. Integr. Cancer Ther..

[52] Medici D., Razzaque M.S., Deluca S., Rector T.L., Hou B., Kang K., Goetz R., Mohammadi M., Kuro-o M., Olsen B.R. (2008). FGF-23-Klotho signaling stimulates proliferation and prevents vitamin D-induced apoptosis. J. Cell Biol..

[53] Andrukhova O., Zeitz U., Goetz R., Mohammadi M., Lanske B., Erben R.G. (2012). FGF23 acts directly on renal proximal tubules to induce phosphaturia through activation of the ERK1/2-SGK1 signaling pathway. Bone.

[54] Bai J.-A., Xu G.-F., Yan L.-J., Zeng W.-W., Ji Q.-Q., Wu J.-D., Tang Q.-Y. (2015). SGK1 inhibits cellular apoptosis and promotes proliferation via the MEK/ERK/p53 pathway in colitis. World J. Gastroenterol..

[55] Chang H.-M., Peng K.-Y., Chan C.-K., Sun C.-Y., Chen Y.-Y., Chang H.-M., Huang C.-L., Liu P.-C., Chen P.-Y., Wang K.-C. (2021). FGF23 ameliorates ischemia-reperfusion induced acute kidney injury via modulation of endothelial progenitor cells: Targeting SDF-1/CXCR4 signaling. Cell Death Dis..

[56] Feng S., Wang J., Zhang Y., Creighton C.J., Ittmann M. (2015). FGF23 promotes prostate cancer progression. Oncotarget.

[57] Kim S.B., Kim J.S., Lee J.H., Yoon W.J., Lee D.S., Ko M.S., Kwon B.S., Choi D.H., Cho H.R., Lee B.J. (2006). NF-kappaB activation is required for cisplatin-induced apoptosis in head and neck squamous carcinoma cells. FEBS Lett..

[58] Li F., Huang L., Su X.-L., Gu Q.-H., Hu C.-P. (2013). Inhibition of nuclear factor-κB activity enhanced chemosensitivity to cisplatin in human lung adeno-carcinoma A549 cells under chemical hypoxia conditions. Chin. Med. J..

[59] Ozkok A., Ravichandran K., Wang Q., Ljubanovic D., Edelstein C.L. (2016). NF-κB transcriptional inhibition ameliorates cisplatin-induced acute kidney injury (AKI). Toxicol. Lett..

[60] Esparza-López J., Medina-Franco H., Escobar-Arriaga E., León-Rodríguez E., Zentella-Dehesa A., Ibarra-Sánchez M.J. (2013). Doxorubicin induces atypical NF-κB activation through c-Abl kinase activity in breast cancer cells. J. Cancer Res. Clin. Oncol..

[61] Wang S., Kotamraju S., Konorev E., Kalivendi S., Joseph J., Kalyanaraman B. (2002). Activation of nuclear factor-kappaB during doxorubicin-induced apoptosis in endothelial cells and myocytes is pro-apoptotic: The role of hydrogen peroxide. Biochem. J..

[62] Mason E.F., Rathmell J.C. (2011). Cell metabolism: An essential link between cell growth and apoptosis. Biochim. Biophys. Acta.

[63] Wallach D., Kovalenko A. (2014). Keeping inflammation at bay. eLife.

